# Interspecific differences in developmental mode determine early cognitive abilities in teleost fish

**DOI:** 10.1007/s10071-023-01828-4

**Published:** 2023-10-13

**Authors:** Giulia Montalbano, Cristiano Bertolucci, Angelo Bisazza, Tyrone Lucon-Xiccato

**Affiliations:** 1 Department of Life Sciences and Biotechnology, University of Ferrara, Via L. Borsari 46, 44121 Ferrara, Italy; 2 Department of General Psychology, University of Padua, Padua, Italy

**Keywords:** Animal cognition, Development, Executive functions, Fish cognition, Individual differences, Self-control

## Abstract

Most studies on developmental variation in cognition have suggested that individuals are born with reduced or absent cognitive abilities, and thereafter, cognitive performance increases with age during early development. However, these studies have been mainly performed in altricial species, such as humans, in which offspring are extremely immature at birth. In this work, we tested the hypothesis that species with other developmental modes might show different patterns of cognitive development. To this end, we analysed inhibitory control performance in two teleost species with different developmental modes, the zebrafish *Danio rerio* and the guppy *Poecilia reticulata*, exploiting a simple paradigm based on spontaneous behaviour and therefore applicable to subjects of different ages. Zebrafish hatch as larvae 3 days after fertilisation, and have an immature nervous system, a situation that mirrors extreme altriciality. We found that at the early stages of development, zebrafish displayed no evidence of inhibitory control, which only begun to emerge after one month of life. Conversely, guppies, which are born after approximately one month of gestation as fully developed and independent individuals, solved the inhibitory control task since their first days of life, although performance increased with sexual maturation. Our study suggests that the typical progression described during early ontogeny in humans and other species might not be the only developmental trend for animals’ cognition and that a species’ developmental mode might determine variation in cognition across subjects of different age.

## Introduction

Cognitive development has been investigated extensively in humans. The general finding is that children show considerable variation in cognitive performance across age. Many cognitive functions are either not detected or highly underdeveloped at birth, and then their performance steadily increases during development (reviewed in Kaye 1986; McCormack 2015; Raghubar et al. 2010; Vasilyeva and Lourenco 2012). Executive functions apparently play a critical role in cognitive development, as a family of functions including working memory and inhibitory control with widespread involvement in various cognitive processes. Executive functions indeed undergo a steady developmental increase (Bedard et al. 2002; Cowan 2014; Diamond 1990; Durston et al. 2002; Pickering 2001; Raghubar et al. 2010; Williams et al. 1999) that has been linked to the improvement in several cognitive processes (e.g., Cowan 2014; Gottwald et al. 2016).

Outside humans, trajectories of cognitive development have received much less attention. This is surprising, considering the number of studies on the cognitive abilities of juvenile individuals in species such as the domestic chick (Lemaire and Vallortigara 2023; Marino 2017; Vallortigara et al. 2010). However, these studies usually investigate subjects of a single age and do not collect longitudinal data (e.g., Wascher et al. 2021). The literature on non-human species contains a relevant number of studies only for one executive function, inhibitory control. Diamond (1990) measured inhibition of the reaching response towards an object placed behind a transparent barrier among rhesus monkeys, *Macaca mulatta*, finding greater performance in 3- to 4-month-old subjects compared to 1- to 2-month-old subjects. Adult dogs inhibited their tendency to approach a food reward behind a transparent barrier 25% more efficiently compared to puppies (Bray et al. 2021). Additionally, a study on ravens, *Corvus corax*, found that chicks improved their inhibitory control performance during a developmental window between 6 and 10 weeks of age (Kabadayi et al. 2017). These findings align with the pattern typically observed in humans, in which inhibitory control emerges toward the end of the first year and undergoes steady development across the toddler stage and preschool years, with incremental improvements that continue throughout childhood, adolescence and early adulthood (Amso and Johnson 2005; Diamond et al. 2007; Ferguson et al. 2021).

Based on these records, one may suggest that the developmental increase of inhibitory control, and potentially several other cognitive abilities, could be a general phenomenon across vertebrates. However, a peculiarity in the development of the species investigated might prevent one from accepting this general idea. Primates display a certain level of altriciality because their new-borns are born undeveloped (Derrickson 1992). Moreover, among primates, humans display a level of altriciality that is considered exceptional: human neonates are totally helpless, and their brain is only 30% of the mass of an adult brain (DeSilva and Lesnik 2006; Rosenberg 2021). While this developmental mode is thought to be an adaptation for growing larger brains later in life, it may hamper brain computational potential during early life (e.g., Chiappa et al. 2018). Similarly, dogs and corvids display a certain degree of altriciality (Lezama-García et al. 2019; Magrath 1990). Reasonably, species with different developmental modes might display different cognitive development trajectories. In particular, early reduced inhibitory control capacities might be a feature only of species in which offspring are born at early developmental stages, such as altricial species.

To investigate the hypothesis that species-specific developmental modes affect early cognitive abilities, we assayed inhibitory control in two teleost fish: the zebrafish, *Danio rerio*, and the guppy, *Poecilia reticulata*. These two species are among the most studied fish in cognitive research (e.g., Bisazza 2011; Lucon-Xiccato 2022; Meshalkina et al. 2017; Oliveira 2013) and are characterised by opposed developmental modes, which was useful for our research question. Zebrafish eggs hatch at the age of 2–3 days post-fertilisation (dpf). Newly hatched larvae mostly sit motionless on the substrate, laying on one side, until they reach the age of 5–6 dpf, when the swim bladder inflates. The early life stages of zebrafish can be compared to those of altricial birds and mammals, because they are undeveloped and unable to perform complex behaviours such as foraging and interacting with conspecifics (Brand et al. 2002; Dreosti et al. 2015). Conversely, guppies are born after a prolonged gestation (approximately 1 month), are immediately active, and display a range of behaviours in the first days of life (e.g., Magurran and Seghers 1990; Romano et al. 2020). Previously, we showed that 10-day-old guppies can solve an inhibitory control task based on a conditioning procedure (Savaşçı et al. 2021), but a lack of similar procedures for zebrafish larvae prevents a direct comparison between the two species. In the current study, we exploited a simpler paradigm based on spontaneous feeding behaviour (Lucon-Xiccato and Bertolucci 2019, 2020) to directly compare inhibitory control of guppies and zebrafish during development. The subjects were exposed to live prey (*Artemia salina* nauplii) sealed, and thus inaccessible, behind a transparent obstacle. To evaluate inhibition, we measured the fish’s ability to withhold capture attempts over time.

Due to difficulty in determining exactly how to match the age of the two species (see ‘Experimental design’ section), we examined zebrafish and guppies separately and we formulated species-specific predictions for each experimental outcome. Based on our hypothesis that a species’ developmental mode affects the development of inhibitory control, we predicted that zebrafish would display increased inhibitory control performance with age (as observed in altricial species of primates), whereas guppies would show efficient inhibitory control early in life.

## Materials and methods

### Experimental design

To analyse inhibitory control performance across development, we aimed to examine groups of fish with different ages for each of the study species. Considering that experience with an inhibitory test may affect performance in subsequent tests (Lucon-Xiccato et al. 2017; van Horik et al. 2018), we opted for a between-subject experimental design. Each subject used in our study was naïve before the experiment and was tested only at one age. Consequently, the age groups were constituted by independent samples.

We measured inhibitory control in zebrafish of three different ages and in guppies of four ages. The age groups were selected based on the zebrafish, because testing this species presented more methodological constraints due to the reduced behavioural repertoire expressed during early development. Zebrafish post-hatching development is typically divided into three main phases: larval, juvenile, and adult phase. We aimed to administer the inhibitory control test to subjects at each of these developmental phases. This was simplified by the fact that in a laboratory population, it is possible to know the day of fertilisation for each subject because adults do not normally reproduce successfully in their maintenance aquaria and a breeding procedure to favour spawning is required (Nasiadka and Clark 2012). For the larval group, an important methodological constraint arises due to the onset of feeding on the prey stimuli. Zebrafish larvae commence to feed on *A. salina* approximately 12 days after hatching (Brand et al. 2002). Results from pilot studies showed that the chosen procedure required a few days of experience with the prey before testing. Thus, the earliest age to start the experiment with the zebrafish was 17 days post-hatching, corresponding to 20 dpf. We then tested one group of zebrafish at 120 dpf, corresponding breeding adult stage, and one group of zebrafish at an intermediate age between hatching and sexual maturation (60 dpf, juvenile stage).

For the guppies, the larval stage is not recognisable and the exact day of fertilisation is difficult to obtain. Guppy new-borns also feed on *A. salina* immediately after birth. Therefore, to match the level of experience with the stimulus prey of larval zebrafish (i.e., 5 days), we assayed guppies’ youngest group at 5 days after hatching. At this age, however, guppies’ general experience with their environment is much less compared to that of zebrafish larvae used in the study. We, therefore, tested another group of guppies at the age of 20 days, comparable to the larvae zebrafish for experience with the environment. Following the logic described for the zebrafish, we then tested one group of guppies at the maturation (120 days) and one group at intermediate age (60 days). Therefore, in the whole study, we assayed three age groups for zebrafish (20, 60 and 120 dpf) and four age groups for guppies (5, 20, 60, and 120 days).

### Subjects

We obtained the zebrafish subjects with a standard breeding protocol, using adults of an outbred wild-type strain maintained at University of Ferrara since 2011 as the breeders. These adult zebrafish were kept in 200 L aquaria under the following condition: water temperature 27 ± 1°C, photoperiod 14:10 h light: dark, water filtered with biological, chemical, and mechanical filters, and food delivered twice per day alternating commercial flakes and *A. salina* nauplii. To induce the spawning, we collected groups of two males and two females from their maintenance aquaria in the late afternoon, and we placed them into two separate sectors of 1.7 L sloping tanks (Tecniplast, Buggiate, Italy). On the next morning, we removed the separation between the two sectors of the sloping tanks, allowing the males and the females to interact and spawn. We then collected the eggs from multiple tanks and held them in Petri dishes (∅ 9 cm; approximately 50 eggs per dish) until hatching, which occurred at 3 dpf. After hatching, a group of healthy larvae was moved into small aquaria filled 1 cm of water (N aquaria = 4; *N* = 10 larvae per aquarium). From 5 dpf onwards, we fed the zebrafish with granular food (Zebrafeed, Sparos, Olhão, Portugal) scaled in size according to their age. At the age of 15 dpf, we fed the fish for the first time with live *A. salina*. After 2 days (age: 17 dpf), we individually moved some of the zebrafish into the experimental apparatuses for the inhibitory control test of the larval stage. The remaining zebrafish were moved into 2 L aquaria (N aquaria = 3; approximately *N* = 10 subjects per aquarium) and raised until the age of 57 dpf for the intermediate testing age (60 dpf). After this point, the remaining group of zebrafish was moved into 60 L aquaria (N aquaria = 2; approximately *N* = 8 fish per aquarium) and raised until maturation to obtain subjects for the last age group (testing at 120 dpf). Fish were maintained in groups before the experiment given that this is a social species, but the testing was conducted individually to avoid interference between subjects. The sample size for the experiment on zebrafish was determined on fish availability and survival: 20 dpf *N* = 9; 60 dpf *N* = 11; 120 dpf *N* = 13.

The guppy subjects were obtained from a domestic strain maintained in our laboratory since 2012. The rearing conditions were as described for the zebrafish. We obtained the subjects by isolating females with abdominal distension and thus, close to parturition, into floating nursery cages. The cages were kept in the rearing aquaria. Each day, we checked for the presence of new-born guppies in the cages. Upon finding new-borns, we immediately moved them into 1-L aquaria (N per aquarium = 10) and fed them granular commercial food and live *A. salina*. After 2 days, some of the guppies were individually moved into the experimental apparatuses and used as subjects of the first age group (testing at 5 days after birth), whereas the remaining guppies were split in three groups. The subjects of one group were individually moved into the testing apparatuses at the age of 17 days for inhibitory control testing at the age 20 days. The subjects of the second group were individually moved into the testing apparatuses at the age of 57 days (testing at 60 days). The fish of the third group were kept into a larger aquarium (60 L; *N* = 10 subjects) since the age of 60 days, and maintained until the last age of testing (120 days). The final sample size for the experiment on guppies was as follows: 5 days *N* = 15; 20 days *N* = 16; 60 days *N* = 11; 120 days *N* = 9. As described for the zebrafish, also guppies’ subjects were maintained in group but then tested individually.

### Inhibitory control test

**Fig. 1:**
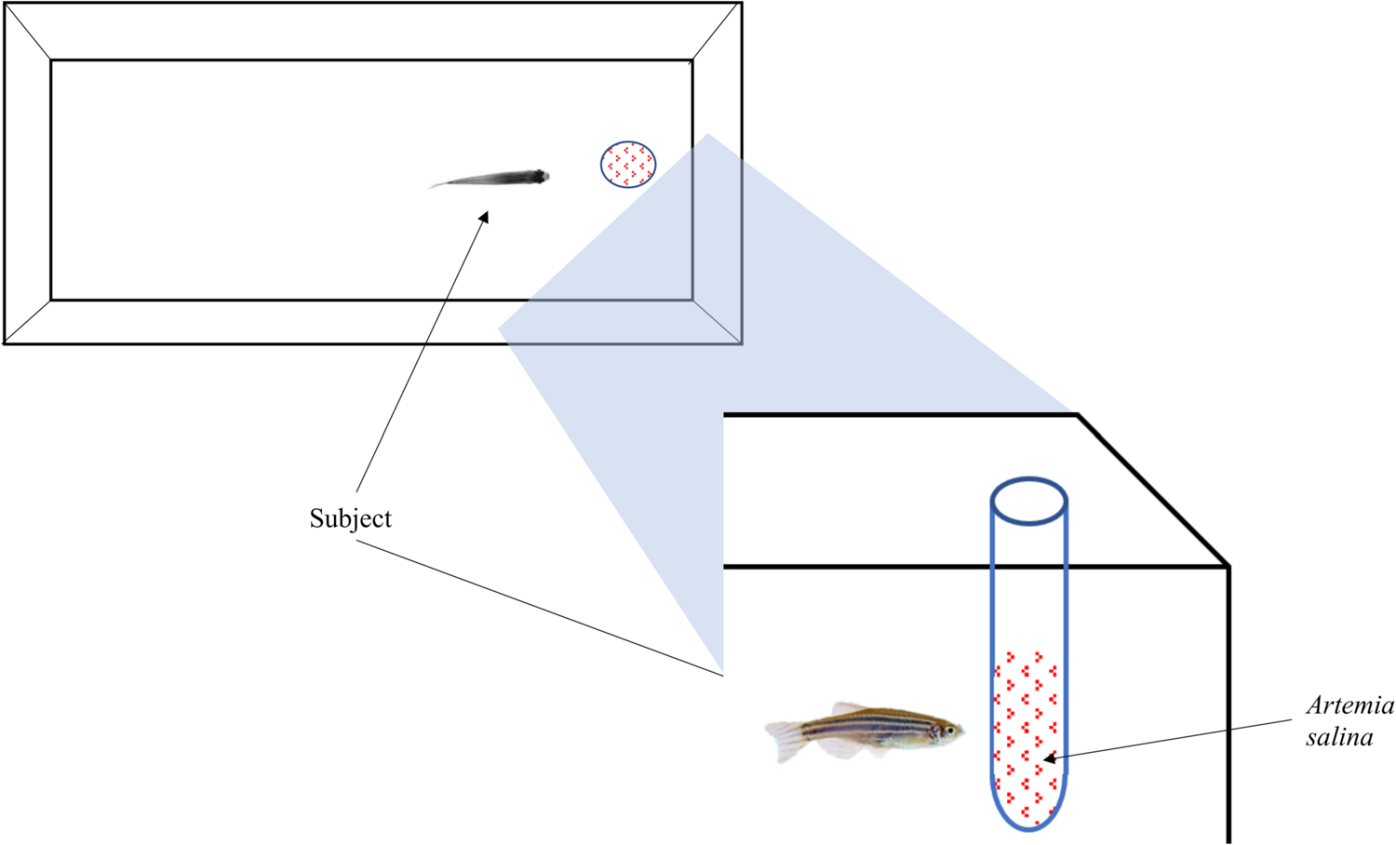
Schematic representation of the apparatus used to assess inhibitory performance in zebrafish. Aerial view (top); lateral view of the front zone (bottom)

To begin the experiment, we moved each individual subject into a rectangular apparatus made of opaque plastic (Fig. 1). We used multiple apparatuses to test several fish simultaneously. The size of the apparatus was scaled according to the body size of the subjects as follow: 15 cm × 5 cm, height 5 cm for the individuals tested up to the age of 60 days (average body length 5–12 mm); 33 cm × 13 cm, height 15 cm, for the adult subjects (average body length 20–25 mm). Above the apparatuses, we installed a white LED strip (Superlight Technology Co. Ltd., Shenzhen, China; illuminance: 700 lx) to provide illumination from 06:00 to 20:00 h. The room was kept at 27 ± 1°C for the entire procedure, ensuring stable water temperature in the apparatuses.

Once in the apparatus, each individual subject underwent an initial pre-test phase, followed by the inhibitory control test. The pre-test phase lasted 3 consecutive days. On each of these days, we fed the subjects with live brine shrimp, *A. salina*, nauplii and commercial food crumbles suspended in water. The quantity of food was adjusted to the size of the subjects to resemble that of normal maintenance conditions. Critically, we consistently released the brine shrimps using a Pasteur pipette in the frontal part of the apparatus to habituate the subject to feed in such area.

The day of testing (day 4), instead of delivering the free food, we inserted a transparent glass tube containing a solution of water and *A. salina* into the apparatus. For the subjects up to the age of 60 days, we used as the tube a standard glass Pasteur pipette with diameter 0.5 cm containing 1 mL of the solution. The tip of the pipette was removed and sealed using a Bunsen burner. For the adults, we used a standard laboratory tube with diameter 1.2 cm containing 4 mL of the solution. After inserting the tube, we recorded the response of the subjects towards the prey in the tube for 20 min using a camera (LEGRIA HF R38, Canon Inc, Oita, Japan) placed above the apparatus.

Playing back the recordings in a computer running the VLC Media Player software (retrieved at https://www.videolan.org), we initially measured the latency to first approach the prey (1 data point per each subject). This variable was used to quantify differences in the motivation to feed. Then, starting from the minute in which the subject first approached the prey, we counted the number of attempts to capture the prey for 10 consecutive minutes with a frame-by-frame analysis. All the subjects approached the prey within 10 min. Given that the maximum recording time was set at 20 min, we obtained 10 min of interaction with the prey for each subject. A capture attempt was scored when the subject touched the glass with its snout.

### Statistical analysis

We conducted the statistical analysis in RStudio version 1.4.1103 (available at https://www.rstudio.com/). We used two-tailed tests and *P* = 0.05 significance level if not stated otherwise. We analysed three dependent variables. The first dependent variable was the initial latency to approach the prey, which was expected to provide an indication of subjects’ motivation to feed. It was compared between age groups using Kruskal–Wallis tests to deal with unequal variances between the groups (detected with the Bartlett test). In case of significant effect of the age, we used Wilcoxon tests with *P* values adjusted with the Holm method to perform pairwise comparisons between each level of the factor. Moreover, we used Spearman’s rank correlation tests to investigate whether the latency correlated with our measure of performance (number of attempts to capture the prey).

The second dependent variable was the number of attempts performed by each subject in each minute of testing. The dependent variable was log-transformed before the analysis to deal with right skewed distribution, also achieving equal variance between age groups. Because the variable included 10 datapoints per subject, we analysed it with repeated measures ANOVAs. The age of the subject and the testing time (from min 1 to min 10) were fitted as fixed effects. For this variable, a significant decrease over time is usually considered indication of inhibition of capture attempts (Lucon-Xiccato and Bertolucci 2019). Therefore, we were mostly interested in detecting significant interactions between age and time, indicating that subjects of different age demonstrated a different rate of inhibition. For the significant interaction in zebrafish data, we ran additional repeated measures ANOVAs, each one fit for the data of each age group, separately. These post hoc ANOVAs allowed us to analyse the decreasing trend in the number of attempts, and therefore inhibitory control, within each age group. Because for guppies the analysis suggested age differences in the average number of attempts, rather than in the decreasing trend of this variable, we ran a one-way ANOVA and a subsequent Tukey post hoc test (computed with the ‘*glht*’ function of the ‘*multcomp*’ R package) on the overall number of attempts performed by each subject to investigate this effect without the confound of repeated measurements. We also conducted a tentative analysis to confirm the species difference with a direct comparison. Because of the experimental design and the developmental biology of the two species, there was no perfect correspondence between the levels of the age factor between zebrafish and guppies. Therefore, we compared separately the younger age and the older age tested in each species with repeated measures ANOVAs. In these models, we were particularly interested in the significance of the species by time interaction, which would indicate a species difference in the inhibition trend.

The third dependent variable was an index of inhibition aimed to describe the change in capture attempts while simultaneously standardising the overall absolute number of attempts across age groups. The index was calculated starting from the minute in which the first approach to the prey occurred (hereafter ‘minute 1’). For any given successive minute ‘X’, the index was calculated as: (N attempts in minute X–N attempts in minute 1)/N attempts in minute 1. This computation provided nine datapoints per subject, which we averaged to obtain a single value per subject. Due do different variances between age groups, we analysed the index using Kruskal–Wallis tests and Wilcoxon test pairwise comparisons with Holm’s adjustment. We also compared the index of inhibition of each age group against zero with Wilcoxon one sample test (an index below zero indicates a decrease of the attempts).

## Results

### Latency to approach the prey

#### Zebrafish

**Fig. 2:**
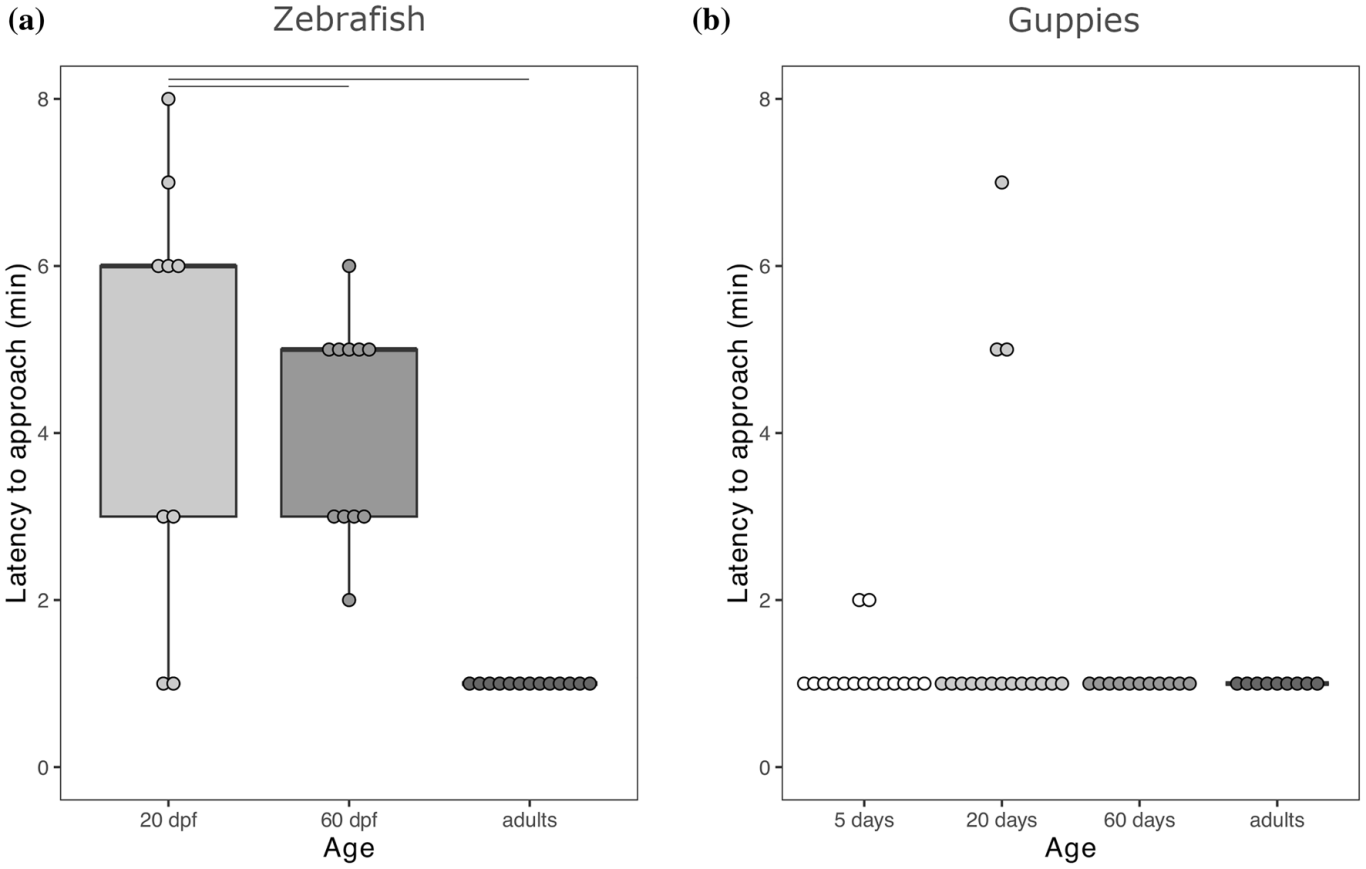
Latency to approach the prey of (**a**) zebrafish and (**b**) guppies from the different age groups. Points represent raw data, internal lines represent medians, box edges represent the first (Q1) and third (Q3) quartiles and whiskers represent Q1 + 1.5 × the interquartile range, IQR, and Q3 + 1.5 × IQR. Bars above the boxes indicate significant differences between age groups revealed by post hoc tests

The Kruskal–Wallis test on the latency to approach the prey of zebrafish showed a significant effect of age (*χ*^2^_2_ = 20.769, *P* < 0.001; Fig. 2a; Table 1). The post hoc tests revealed that the adult zebrafish were significantly faster to approach the prey as compared to the larval (*P* < 0.001; Fig. 2a) and the juvenile zebrafish (*P* < 0.001; Fig. 2a). There was no significant difference in latency to approach the prey between the larval and the juvenile zebrafish (*P* = 0.459; Fig. 2a). The latency to approach did not correlate with the number of attempts to capture the prey (*ρ* = 0.048, *P* = 0.790).

**Table 1:** Descriptive statistics (means ± standard deviation) of the latency to approach the prey and the inhibition index of zebrafish and guppies divided by age group

Species, variable	5 days	20 days	60 days	120 days
Zebrafish, latency to approach	–	4.56 ± 2.60 min	4.09 ± 1.30 min	1.00 ± 0.00 min
Zebrafish, inhibition index	–	0.75 ± 1.15	0.13 ± 0.94	− 0.56 ± 0.27
Guppy, latency to approach	1.13 ± 0.35 min	1.88 ± 1.93 min	1.00 ± 0.00 min	1.00 ± 0.00 min
Guppy, inhibition index	− 0.16 ± 0.56	− 0.20 ± 0.23	− 0.35 ± 0.37	− 0.91 ± 0.07

#### Guppies

The Kruskal–Wallis test on the latency to approach the prey of guppies showed no significant effect of age (*χ*^2^_3_ = 3.907, *P* = 0.272; Fig. 2b; Table 1). The latency to approach did not correlate with the number of attempts to capture the prey (*ρ* = 0.048, *P* = 0.740).

### Number of attempts to capture the prey

#### Zebrafish

The repeated measures ANOVA on the number of capture attempts of zebrafish revealed a significant interaction between age and testing time (F_2,294_ = 8.407, *P* < 0.001). The main effect of time was also significant (F_1,294_ = 28.629, *P* < 0.001), but the main effect of age was not significant (F_2,30_ = 0.636, *P* = 0.536).

**Fig. 3:**
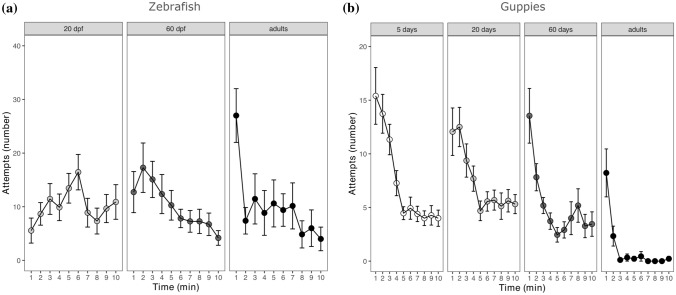
Number of capture attempts observed in (**a**) zebrafish and (**b**) guppies from the different age groups across the 10 min of observation. The points indicated means and the error bars indicated standard errors. Note that the scale differs between the two panels

By analysing separately subjects with different age, we found a significant decrease in the number of capture attempts over time in adult zebrafish (F_1,116_ = 31.777, *P* < 0.001; Fig. 3a) and in juvenile zebrafish (F_1,98_ = 12.398, *P* < 0.001; Fig. 3a). However, the larval zebrafish did not show this decrease in the number of capture attempts (F_1,80_ = 0.364, *P* = 0.548; Fig. 3a).

#### Guppies

The repeated measures ANOVA on the number of capture attempts of guppies revealed a significant main effect of time (F_1,455_ = 156.533, *P* < 0.001) and a significant main effect of age (F_3,47_ = 34.259, *P* < 0.001). The interaction between age and time was not significant (F_3,455_ = 1.4745, *P* = 0.221). This pattern of results indicated that guppies of different ages decreased the number of attempts over time and they did so with the same rate (Fig. 3b). The one-way ANOVA on the sum of attempts performed by each subject confirmed the main effect of age (F_3,47_ = 34.392, *P* < 0.001). The Tuckey post hoc test run on this model indicated that the adult guppies performed less attempts (11.89 ± 7.69 attempts, mean ± standard deviation) compared to guppies of all the other ages tested (5 days: 73.80 ± 34.12 attempts, *t* = 9.206, *P* < 0.001; 20 days: 73.63 ± 29.92 attempts, *t* = 9.127, *P* < 0.001; 60 days: 51.55 ± 19.40 attempts, *t* = 6.985, *P* < 0.001). There were no significant differences in the remaining age comparisons (5 days versus 20 days: *t* = 0.172, *P* = 0.996; 5 days versus 60 days: *t* = 1.869, *P* = 0.254; 20 days versus 60 days: *t* = 0.188, *P* = 0.337).

#### Comparison between the two species

The model that compared the two species at the younger age found a significant main effect of time (F_1,214_ = 34.297, *P* < 0.001) but no significant main effect of species (F_1,22_ = 0.384, *P* = 0.542). Critically, the interaction was significant (F_1,214_ = 29.816, *P* < 0.001), confirming that only the guppies showed inhibition.

The model on the adult subjects, found significant main effects of species (F_1,20_ = 13.118, *P* = 0.002) and time (F_1,196_ = 65.295, *P* < 0.001). The interaction was not significant (F_1,196_ < 0.001, *P* = 0.992), indicating that both species showed inhibition at this age.

### Index of inhibition

#### Zebrafish

**Fig. 4:**
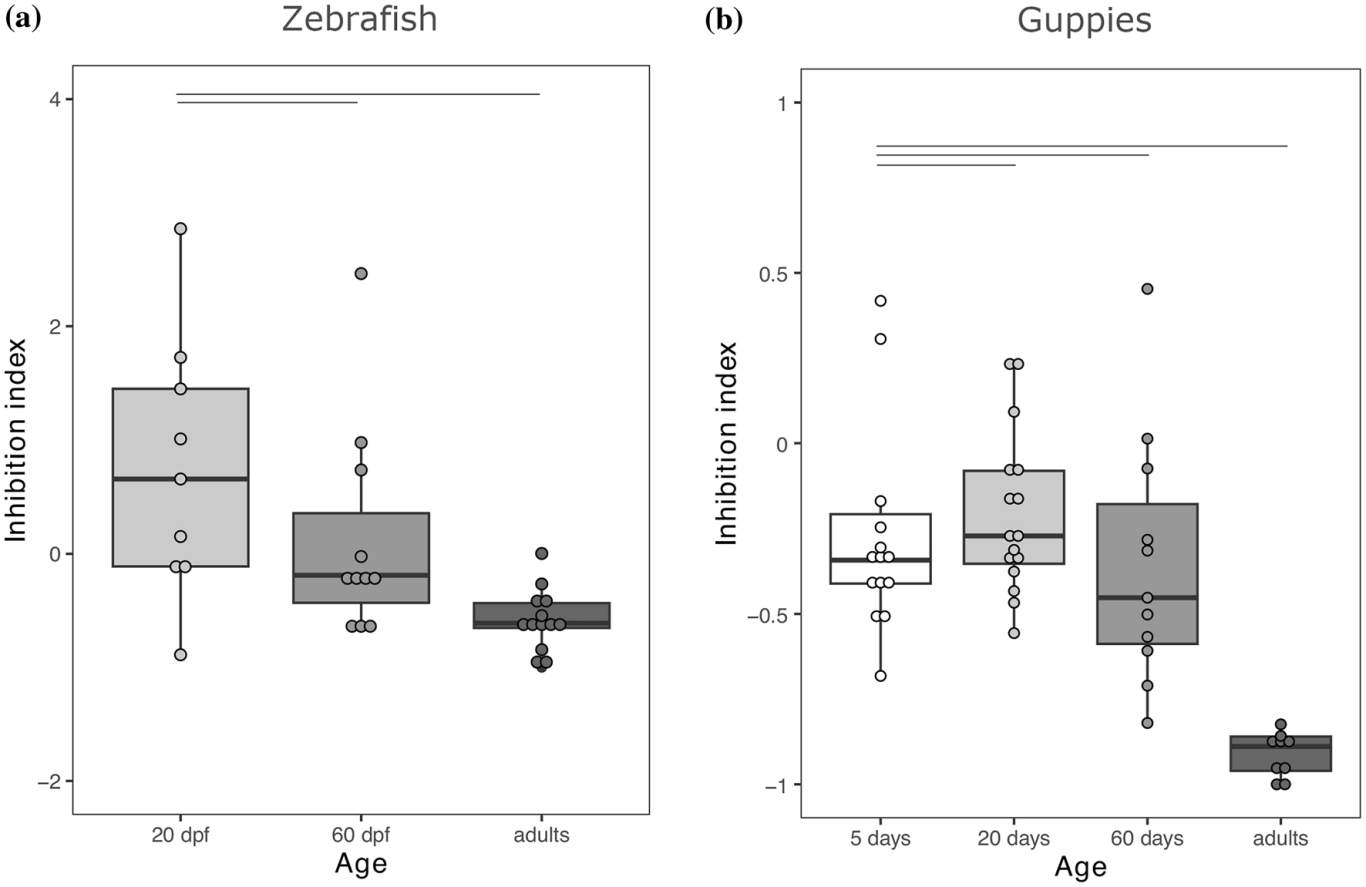
Index of inhibition calculated from the change in the capture attempts of the different age groups of (**a**) zebrafish and (**b**) guppies. Points represent raw data, internal lines represent medians, box edges represent the first (Q1) and third (Q3) quartiles and whiskers represent Q1 + 1.5 × the interquartile range, IQR, and Q3 + 1.5 × IQR. Bars above the boxes indicate significant differences between age groups revealed by post hoc tests. Note that the scale differs between the two panels

The Kruskal–Wallis test on the index of inhibition of zebrafish showed a significant effect of age (*χ*^2^_2_ = 11.951, *P* = 0.003; Fig. 4a; Table 1). The post hoc tests revealed that the index of inhibition was significantly lower in adult zebrafish compared to larvae (*P* = 0.004) and compared to juveniles (*P* = 0.031). There was no significant difference in the comparison between larvae and juveniles (*P* = 0.112). When comparing the index of inhibition against zero, we found that it was significantly negative for the adults (*P* < 0.001), not significantly different from zero for juveniles (*P* = 0.831) nor larvae (*P* = 0.098).

#### Guppies

The Kruskal–Wallis test on the index of inhibition of guppies found a significant difference between subjects of different age (*χ*^2^_3_ = 23.524, *P* < 0.001; Fig. 4b; Table 1). The post hoc tests revealed that the index of inhibition was significantly lower in adult zebrafish (versus 5 days: *P* < 0.001; versus 20 days: *P* < 0.001; versus 60 days: *P* < 0.001). There was no significant difference in the remaining comparisons (all *P* values > 0.4). When comparing the index of inhibition against zero, we found that it was significantly negative for the adults (*P* = 0.009), 20-day old guppies (*P* = 0.005), and 60-day old guppies (*P* = 0.018), but this failed to meet significance for 5-day old guppies (*P* = 0.095).

## Discussion

Our experiments suggested a difference in the developmental trajectory of inhibitory control between two teleost fish with different developmental modes. The species with a developmental mode similar to altriciality, the zebrafish, failed to solve our inhibitory control task soon after hatching, and then improved during ontogenesis. Indeed, the youngest group of zebrafish (larval stage) did not show a decreasing trend in the number of attempts to capture the prey behind the transparent barrier and had an index of inhibition in the positive direction, rather than negative as expected in case of inhibition. Conversely, our study species with the developmental mode similar to precocial mammals or birds, the guppy, displayed evidence of inhibitory control in terms of significantly decreased capture attempts and although not reaching significance, a negative inhibition index immediately after being born. Guppies also showed a marked improvement in inhibitory performance at sexual maturation, evidenced by a lower average number of capture attempts and lower index of inhibition as adults. This may be related to the sex difference favouring females in inhibitory performance previously reported in guppies (Lucon-Xiccato and Bisazza 2017; Lucon-Xiccato et al. 2020).

For zebrafish, but not guppies, we also found an age effect on the latency to approach the prey. This effect could be due to age differences in motivation, and particularly to lower motivation, as indicated by higher latency, in the larval zebrafish. However, lower motivation should be accompanied by earlier reduction in capture attempts in the larval zebrafish (van Horik et al. 2018), which we did not observe (Fig. 3a). Moreover, we did not find evidence that the latency to approach correlated with interaction with the prey. Therefore, the most likely explanation for our data is that larval zebrafish took longer to reach the stimulus prey due to locomotory or sensory limitations. Interestingly, there appears to be a species difference in latency to approach the stimulus, at least for immature subjects (Fig. 2). This observation indicates that the juveniles of the two species may react differently to the experimental setting, as previously observed for adults in other inhibitory control settings (Santacà et al. 2019), suggesting the need for caution when comparing species also during development.

Evidently, interspecific developmental and behavioural differences prevented us from precisely matching the testing age between our two study species. For instance, guppies could not be tested shortly after fertilisation, as zebrafish could, simply because the guppies were still to be born. Similarly, we could not test zebrafish immediately after hatching because at that time, they do not show the foraging behaviour required for the task. Even if a perfect match were possible for these parameters, it would not be possible to exclude confounding effects due to interspecific differences in the pace of developmental processes, which can vary substantially even between species belonging to the same genus (Diaz-Cuadros et al. 2023; Martin and Schwabl 2008; Krasnov et al. 2001). The difficulty of matching all the parameters is an issue that deserves careful evaluation in comparative studies on cognitive development, not only in our study system.

We selected the testing age of the two species by balancing between the various factors and assaying multiple early age points in one of the two species. As a result, we believe that even in spite of the aforementioned comparative difficulties, our findings are clear on the fact that only the species with a developmental mode similar to precociality, the guppy, displays evidence of inhibitory control immediately after birth. Therefore, with the limitation of being based on only two species, our study seems to support the idea that cognitive development might vary according to the developmental mode of the species. It will be important to confirm this interpretation with comparative studies on other species. While doing so, factors beside the developmental mode should be considered. For instance, in the jack mackerel, *Trachurus japonicus*, ontogenetic improvement in several cognitive tasks, including one aimed to assess an executive function, has been reported in correspondence of a major ecological shift that occurs during development (Takahashi et al. 2010, 2012, 2014). Therefore, not only interspecific variability in the developmental mode, but also in ecological factors, might substantially affect the developmental trajectories of cognitive functions. Developmental changes in predation risk have been reported for both study species (Mattingly and Butler 1994; Spence et al. 2008). Zebrafish also show developmental changes in social behaviour (Buske and Gerlai 2011). It is worth investigating these ecological changes to test hypotheses on adaptive age variation in inhibitory control (Amici et al. 2008; Lucon-Xiccato et al. 2022; Ryer and Olla 1991).

Another research question raised by our study pertains to whether the developmental differences between species might be present in other cognitive traits. This may occur for two reasons. First, executive functions such as the inhibitory control are potentially involved in various aspects of individuals’ cognitive performance (Beran and Hopkins 2018; Kralik et al. 2002). This means that the developmental changes in inhibitory control likely affect the efficiency of other cognitive traits. Second, other cognitive traits may have distinct developmental trajectories, independent from executive functions. Some useful, albeit indirect, information on the latter possibility may be available for fish, for instance, in studies on numerical abilities in guppies and zebrafish (Bisazza et al. 2010 and Sheardown et al. 2022, respectively). The study with guppies showed an innate capacity to discriminate between social groups differing by one individual up to three vs four units (0.75 ratio), the same numerical acuity shown by adults tested in similar conditions (Bisazza et al. 2010). Zebrafish tested between age 21 and 33 dpf and showed an age effect in the most difficult comparison, two versus three (0.67 ratio), which was discriminated only by the oldest larvae (Sheardown et al. 2022). Although in this case, the cognitive ability under investigation (i.e. numerical discrimination) seems to be present at birth in both species (Bisazza et al. 2010; Lucon-Xiccato et al. 2023), it apparently increases with age only in the zebrafish.

One last aspect that deserves to be integrated in the comparative analysis of cognitive development concerns proximate mechanisms. For instance, failure to solve the inhibitory control task by young zebrafish might be determined by neural structures that are still immature. A study in an invertebrate, the cuttlefish *Sepia*, has associated ontogenetic improvement in a task similar to that of the present study with the maturation of the vertical lobe complex in the brain (Dickel et al. 2001). Using various paradigms, a number of neural circuities have been associated with inhibition in humans (reviewed in Parker et al. 2013), and at least one of them, the ventral fronto-striatal circuitry, undergoes developmental maturation that has been associated with the progression of inhibitory control (Durston et al. 2002). Putative homologous regions of the ventral fronto-striatal circuitry have been identified in zebrafish (Parker et al. 2013), calling for analysis of their maturation as determinant of inhibitory control development.

## Data Availability

Data will be provided upon direct request.
